# Suitability of point kernel dose calculation techniques in brachytherapy treatment planning

**DOI:** 10.4103/0971-6203.62202

**Published:** 2010

**Authors:** Thilagam Lakshminarayanan, K. V. Subbaiah, K. Thayalan, S. E. Kannan

**Affiliations:** Safety Research Institute, Atomic Energy Regulatory Board, Kalpakkam - 603 102, India; 1 Barnard Institute of Radiology & Oncology, Madras Medical College & Government General Hospital, Chennai - 600 003, India

**Keywords:** Brachytherapy, cervical cancer, low dose rate, treatment planning system, point kernel code, Monte Carlo

## Abstract

Brachytherapy treatment planning system (TPS) is necessary to estimate the dose to target volume and organ at risk (OAR). TPS is always recommended to account for the effect of tissue, applicator and shielding material heterogeneities exist in applicators. However, most brachytherapy TPS software packages estimate the absorbed dose at a point, taking care of only the contributions of individual sources and the source distribution, neglecting the dose perturbations arising from the applicator design and construction. There are some degrees of uncertainties in dose rate estimations under realistic clinical conditions. In this regard, an attempt is made to explore the suitability of point kernels for brachytherapy dose rate calculations and develop new interactive brachytherapy package, named as BrachyTPS, to suit the clinical conditions. BrachyTPS is an interactive point kernel code package developed to perform independent dose rate calculations by taking into account the effect of these heterogeneities, using two regions build up factors, proposed by Kalos. The primary aim of this study is to validate the developed point kernel code package integrated with treatment planning computational systems against the Monte Carlo (MC) results. In the present work, three brachytherapy applicators commonly used in the treatment of uterine cervical carcinoma, namely (i) Board of Radiation Isotope and Technology (BRIT) low dose rate (LDR) applicator and (ii) Fletcher Green type LDR applicator (iii) Fletcher Williamson high dose rate (HDR) applicator, are studied to test the accuracy of the software. Dose rates computed using the developed code are compared with the relevant results of the MC simulations. Further, attempts are also made to study the dose rate distribution around the commercially available shielded vaginal applicator set (Nucletron). The percentage deviations of BrachyTPS computed dose rate values from the MC results are observed to be within plus/minus 5.5% for BRIT LDR applicator, found to vary from 2.6 to 5.1% for Fletcher green type LDR applicator and are up to −4.7% for Fletcher-Williamson HDR applicator. The isodose distribution plots also show good agreements with the results of previous literatures. The isodose distributions around the shielded vaginal cylinder computed using BrachyTPS code show better agreement (less than two per cent deviation) with MC results in the unshielded region compared to shielded region, where the deviations are observed up to five per cent. The present study implies that the accurate and fast validation of complicated treatment planning calculations is possible with the point kernel code package.

## Introduction

The curative potential of radiation therapy in the management of gynecological cancers is greatly enhanced by the use of intracavitary brachytherapy (ICBT). ICBT is a method of delivering dose in a localized way. The most common brachytherapy techniques used in the treatment of cervical carcinoma are based on the Manchester source system[1–3] comprising an intrauterine (IU) tube and two vaginal ovoids. This arrangement produces the classical “pear-shaped” isodose distribution with the widest part of the distribution located around the cervix.

To protect rectum, bladder and vaginal regions various lead or tungsten shields are employed in ovoids of some cervical and vaginal applicators. Some applicators incorporate shields with special geometry, which do not have cylindrical symmetry. It is quite complex to take this correction into account. Many treatment planning systems neglect source and applicator encapsulations and colpostat shielding in their dose calculation algorithms. They perform dose computation either by simple superposition or by interpolations from a table of dose rates in water, stored for each source. These dose rate tables (DRT) assume cylindrical symmetry of the sources and the medium in which the calculations is made is water equivalent, with no modification for different heterogeneities and no account for inter-source effects or applicator attenuation. For typical clinical applications, the dose calculation by simple superposition or using DRT, which accounts only for the contributions of individual sources and the source distribution, may overestimate the dose at ICRU-38 reference points.[4] In the recently developed commercially available treatment-planning systems, the AAPM Task Group 43 (TG-43) protocol[5,6] is recommended for dose rate calculation. The TG-43 approach consists of using measured and MC generated dose rate distributions directly for clinical dose calculations. In the present study, a new dose rate calculation technique is proposed for brachytherapy treatment planning.

A point kernel code BrachyTPS, developed for computing dose rate distributions by taking into account the effect of heterogeneities in ICBT applicators, is presented. The code computes dose rates at the desired locations due to point or volumetric radioactive sources in the presence of other non-emitting material acting as shielding. Buildup factors are considered to account for radiation scattering effects, using the geometric-progression (GP) formula in the fitting function. The dose computations have been performed by treating each individual volumetric source as several point sources and by summing, at the point of interest, the contributions of the individual sources placed inside the applicator. The major advantage of this code is that the heterogeneity in brachytherapy is taken care by using two regions buildup factors, proposed by Kalos.[7]

The purpose of the present work is to evaluate the computational efficiency of the point kernel code with the dose calculation algorithm accounting for source encapsulation, applicator attenuation and the shielding effects of the ICBT applicators. The accuracy of the code has been tested by performing dose rate computations for some of the commonly used ICBT applicators and by comparing with estimates obtained from one of the most precise methods of brachytherapy dose calculations like MC simulations and with the other available literatures.[8–10] Results of these comparisons are presented and discussed in the following sections.

## Materials and Methods

MC simulations are generally reliable for dose rate computations, especially in brachytherapy, provided that the geometrical modeling, input information and photon interaction cross-section data etc. are accurate. But, owing to its slow computational time it is not a choice in clinical dosimetry. As a solution to this problem a point kernel based technique has been developed to meet the clinical requirements in precision and time. As primary input data, the code takes patients' planning data including the source specifications, dwell positions, dwell times and it computes the dose rates at reference points by dose point kernel formalisms, with multi-layer shield build-up factors accounting for the contributions from scattered radiation. In the present study, MC simulations are also performed for all cases to test the accuracy of BrachyTPS computations.

### BrachyTPS – Interactive Point kernel Code Based Treatment Planning Package:

#### A point kernel ray tracing technique for dose rate computations

For dose calculations, the BrachyTPS code uses the point-kernel ray tracing technique. In this method, the point kernel representing the transfer of energy by the uncollided flux along a line-of-path is combined with an appropriate buildup factor to account for the contribution of scattered photons. For the distributed volume source case, the point kernel is integrated over the source volume for all probable energies emitted. The dose rate D˙(r)can be represented as an integral equation

(1.0) $$\text{D}˙\left(\text{r}\right)=\text{k}\int_{\text{v}}\frac{\text{S}\left(\text{r'}\right)\text{B}\left(\text{μ|r−r'|},\text{E}\right)\exp\left(-\text{μ}|\text{r}-\text{r'|}\right)\text{dV}}{4\prod {|\text{r}-\text{r'|}}^{2}}$$

Where, K flux-to-dose rate conversion factor[11]

S(r') source density in Bq /cm^3^

B (*μ*|r−r'|, E) Dose buildup factor at gamma ray energy E

*μ* Gamma ray linear attenuation coefficient at energy E

|r−r'| Distance between the detector and source point

The above integral is replaced by summation by discretizing the source in to small pieces of definite small volume. For calculations, point source located at the center of volume element with the source strength present in that volume is considered. Line sources are approximated as series of point sources with Δl is equal to 0.05mm. The number of point sources assumed depends on the length of the line source. The optical distance from the point source to the detector location is calculated by the Combinatorial Geometry (CG) package. By substituting appropriate values of the variables in equation (1.0), the dose rate is calculated. The dose rates thus estimated are summed up over the entire region of the source and over all possible gamma ray energy groups. The buildup factors used are based on the ANSI/ANS- 6.4.3-1991,[12] which includes buildup factors computed through gamma-ray transport calculations performed in infinite homogeneous media from various codes.

Buildup factors account for the estimation of scattered contribution to the dose rate and thus avoiding detailed radiation transport calculations. The code uses GP functional coefficients available for 26 commonly used shielding materials in the energy range of 15keV to 15 MeV. The functional form of Harima *et al*,[13] for computing buildup factor is

(2.0) $$\begin{array}{llll} \text{B}\left(\text{x}\right) & = & 1+\left(\text{b}-1\right)*\left({\text{k}}^{\text{x}}\text{−}1\right)/\left(\text{k−1}\right) & \text{for k}\neq \text{1}\\ & = & 1+\left(\text{b−1}\right)*\text{x} & \text{for k}=1 \end{array}$$

(3.0) $$\text{K}\left(\text{x}\right)={\text{C*x}}^{\text{a}}+\text{d*}\left[\frac{\tanh\left({\text{x/x}}_{\text{k}}-2\right)\text{−}\tanh\left(\text{−2}\right)}{1\text{−}\tanh\left(\text{−2}\right)}\right]$$

Where,

B is Buildup factor at 1 mean-free-path

x = (*μ*|r−r'|) is the source to detector distance in mean-free-path

a, b, C, d and x_K_ are parameters built in to the code as a fixed blocked data.

The present study employs a method proposed by Kalos,[7] for computing multi-layer shield build-up factors. For two-layer shields of optical thicknesses *l*_1_and *l*_2_ and effective atomic numbers *Z*_1_ and *Z*_2_, numbered in the direction from source to detector, a commonly applied rule is that if *Z*_2_ >*Z*_1_, then the overall buildup factor is approximately equal to the buildup factor *B*_2_ for material 2 evaluated at the total optical thickness (*l*_1_ + *l*_2_). On the contrary if *Z*_1_ >*Z*_2_, then the overall buildup factor is the product *B*_1_(*l*_1_) × *B*_2_(*l*_2_).

But a more precise method for two layer shields is that of Kalos,[7] which states that the overall buildup factor for two-regions of optical thickness *l*_1_ and *l*_2_ and effective atomic numbers *Z*_1_ and *Z*_2_, numbered in the direction from source to detector, is

(4.0) $$B=B_{2}\left(l_{2}\right)+\frac{B_{1}\left(l_{1}\right)-1}{B_{2}\left(l_{1}\right)-1}\left[B_{2}\left(l_{1}+l_{2}\right)-B_{2}\left(l_{2}\right)\right]\text{ for }Z_{1}>Z_{2}$$

(5.0) $$B=B_{2}\left(l_{2}\right)+\left[B_{2}\left(l_{2}+l_{2}\right)-B_{2}\left(l_{2}\right)\right]\times \{\frac{B_{1}\left(l_{1}\right)-1}{B_{2}\left(l_{1}\right)-1}\exp\left(-1.7l_{2}\right)+\frac{{\left({\text{μ}}_{\text{C}}/\text{μ}\right)}_{1}}{{\left({\text{μ}}_{\text{C}}/\text{μ}\right)}_{2}}\left[1-\exp\left(-l_{2}\right)]\right\}\text{ for }Z_{2}>Z_{1}$$

The Brachytherapy dosimetry, which usually involves two distinct regions viz. source with encapsulation and tissue medium, can be treated using equation 4.0 of Kalos's method and is employed in BrachyTPS package.

#### Isodose distributions

**Figure 1:**
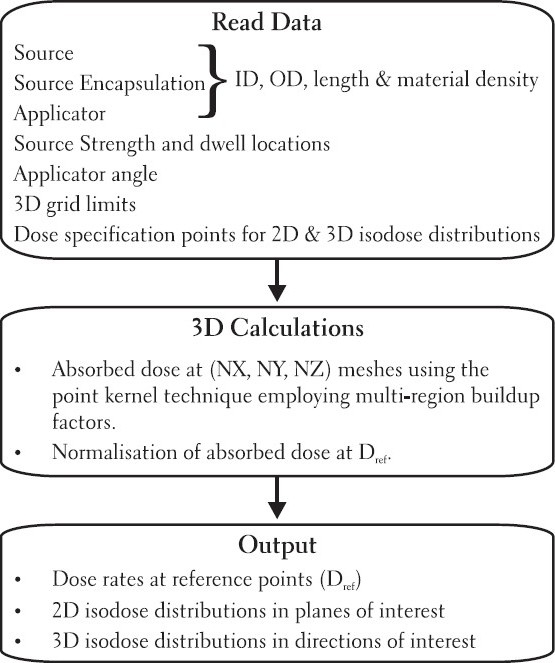
Block diagram of BrachyTPS for calculating 2D and 3D isodose distributions

The developed package computes three dimensional dose distributions in the 3D matrix defined by user selection of calculation limits, which determines thereby the resolution of the calculation grid. This approach allows us to evaluate the isodose curves produced by the sources, in any plane, normalized at any point of interest and a three dimensional (3D) distribution of user selected isodose surfaces in any direction. The block diagram of BrachyTPS for calculating 2D and 3D isodose distributions is shown in Figure 1.

#### Layout of BrachyTPS

**Figure 2:**
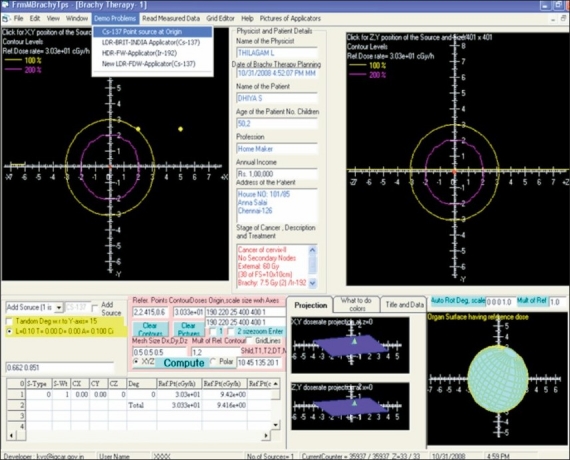
Layout of Graphical user Interface (GUI) developed for BrachyTPS

The layout of the developed interactive point kernel package BrachyTPS is shown in Figure 2. The options and controls used to feed the input information like source and applicator specifications, mesh sizes, reference points for computations, reference contour levels and to get the output information like dose rates at reference points and 2D and 3D isodose distributions are displayed in it. As a demo problem, the 2D and 3D isodose distributions computed around a ^137^Cs point source using BrachyTPS package are shown in Figure 2.

#### Description of Applicators with Respective Sources

The BRIT LDR applicator with BRIT made CSA-1 and CSA-2 ^137^Cs sources, the Fletcher Green type LDR applicator with Amersham made CDC-J ^137^Cs source and Fletcher-Williamson HDR applicator with Nucletron made mHDR ^192^Ir are studied in the present work. These applicators consist of an intrauterine tube and tilted cylindrical vaginal ovoids. The intrauterine tubes of are made up of stainless steel and are either straight or bent anteriorly at angles of 15°, 30° or 45°. The vaginal ovoids are also made of stainless steel and have two bends. The bend in the plane of uterine tube helps to increase the separation between vaginal sources and the bend in the sagittal plane helps to position the vaginal ovoids at an angle to the plane of the uterine sources so as to minimize the dose to the rectum. As an example of shielded applicator, the dose rate distribution taking into account the effect of the internal structure as well as the shielding used with a vaginal cylindrical applicator set (Applicator 084.320, Nucletron Corporation, Columbia MD, USA) with Nucletron made mHDR ^192^Ir is estimated. The vaginal cylinder consists of 15-cm long cylinder plastic shell with an outer diameter of 3.0 cm and 0.5 cm thick wall. It centered on a thin-wall stainless steel tube (0.4 cm outer diameter) which serves as pathway for the ^192^Ir source. There is an air gap between the steel tube and the inner wall of the cylinder. This gap can be filled with 0.8 cm thick tungsten 90°, 180°, or 270° shields.[10,14] All these components are held in place along with the central tube by the plastic end cap. The mHDR ^192^Ir source of strength 1Ci (3.7×10^4^ MBq) is simulated approximately 4.5 cm from the tip of the applicator. MCNP plot depicting the geometrical models of the shielded vaginal cylinder is shown in Figure 3b.

The main features of all these applicators and their respective sources are given in Table 1. In MC simulations, the applicators and the sources are modeled with the exact geometry as described in previous literatures[8–10,15–21] but in BrachyTPS simulations, computations are performed with the homogenized density of material for the given dimensions.

**Table 1:** Main features of the applicator and their respective sources

*Applicator*	*Source*	*Source Information*				*Applicator Information*		
			
		*Active*		*Encapsulation*		*ID (cm)*	*OD (cm)*	*Applicator material & density (g/cc)*
						
		*Diameter (cm)*	*Length (cm)*	*Outer Diameter – OD (cm)*	*Material & density (g/cm^3^)*						
BRIT (LDR)	BRIT CSA-1 ^137^Cs	0.18	1.5	0.3	SS, 8.02	0.5	0.6	SS, 8.02
	BRIT CSA-2 ^137^Cs	0.18	1.0	0.3	SS, 8.02	0.5	0.6	SS, 8.02
Fletcher Green type (LDR)	Amersham CDC-J ^137^Cs	0.165	1.35	0.265	80% Pt 20% Fe, 21.644	0.4	0.6	SS, 8.02
Fletcher-Williamson(HDR)	Nucletron mHDR ^192^Ir	0.065	0.36	0.09	SS,8.02	0.3	0.4	SS, 8.02
Vaginal Cylinder	Nucletron mHDR ^192^Ir	0.065	0.36	0.09	SS,8.02	2.5& 0.3	3.0& 0.4	Plastic shell & SS tube

#### MCNP - Monte Carlo Method

MC simulations are performed using well established Monte Carlo N-Particle (MCNP-4B) code[18] developed at LANL. MCNP is a general purpose continuous energy, generalised-geometry Monte Carlo code, which deals with transport of neutrons, photons, and coupled electron photon transport, i.e., transport of secondary electrons resulting from gamma interactions. The applicators are modeled with the respective sources as per the material and geometrical information available in the previous studies.[8–10,15–21] MC plots of source loadings in BRIT LDR applicator with long, straight intrauterine tube and vaginal ovoids (of angulation ~−40°) at 3 cm separation is shown in Figure 3a and Figure 3b depicts the geometrical models of the shielded vaginal cylinder as simulated in MC code. The deviations observed between MCNP simulations of ^137^Cs sources with and without Ba K X-rays are less than 0.5% and therefore, photon of energy 0.662MeV alone is considered with the corresponding probability of emission (0.851) for all MCNP simulations using ^137^Cs sources. The gamma ray photon energies and corresponding emission probabilities for the ^192^Ir isotope is taken from literature,[10,14] for simulation of the HDR brachytherapy source. The gamma photon emission spectrum of ^192^Ir is shown as a line spectrum representation in Figure 4. The energy distribution of the photon source used for this study is realized using the SDEF card options of the MCNP viz. SI, SP cards. All MCNP calculations are carried out with Mode P and photon energy cutoff has been set to one keV.

**Figure 3a:**
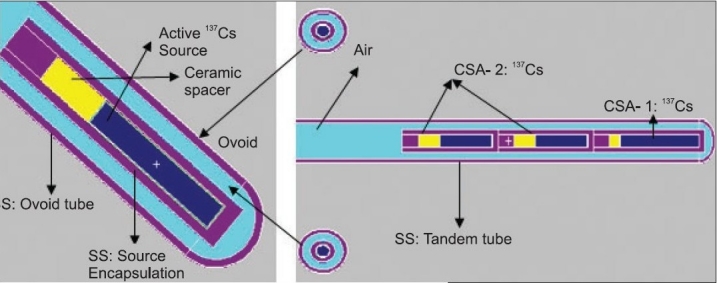
MCNP plots of source loadings in BRIT LDR applicator with long, straight intrauterine tube and vaginal ovoids (of angulation ~−40°) at 3.0 cm separation

**Figure 3b:**
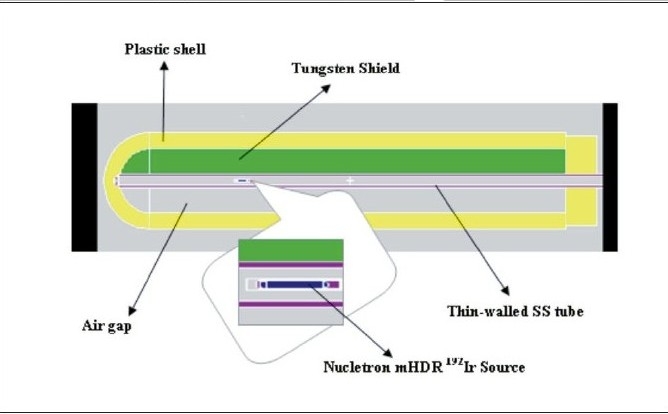
MCNP plot of geometric models of the shielded vaginal cylinder

**Figure 4:**
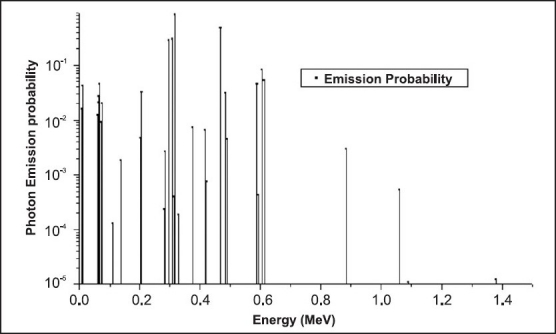
Photon emission line spectrum of ^192^Ir HDR source as function of energy

The MCNP tally option utilized for this problem is the *F5 (MeV/cm^2^) tally with Mode P, to evaluate the required parameter of interest viz. dose rates. The *F5 tally of MCNP code estimates photon energy fluence spectrum for one starting particle per second. The photon energy fluence spectrum thus obtained using *F5 tallies of MCNP simulations are converted into dose rates (Gy/hr) by using the mass energy absorption coefficients of water provided by Hubbell and Seltzer[19] and by using the corresponding conversion factors as depicted in the following equation

(6.0) $$\begin{array}{lll} & & \text{A}(\text{J/MeV}))*\text{T}\left(s/h\right)*\text{D}\left(particles/s\right)\\ Dose\;rate\left(Gy/h\right) & = & \frac{*\sum_{E_{\min}}^{E_{\max}}\text{ψ}\left(E_{i}\right)*\left(\frac{{\text{μ}}_{en}\left(E_{i}\right)}{\text{ρ}}\right)\text{X}\Delta \text{E}\left(MeV/g\right)}{C\left(kg/g\right)} \end{array}$$

Where E_i_ is Midpoint of each energy bin, ψ(E_i_) is the differential photon energy fluence (MeVcm^−2^) per unit energy interval at energy E_i,,_ per source photon and ΔE is the energy bin width $\left(\frac{{\text{μ}}_{en}\left(E_{i}\right)}{\text{ρ}}\right)$. is the mass energy absorption coefficient of water at energy E_i_. It may be noted that

(7.0) $$\text{ψ}\left({\text{E}}_{\text{i}}\right)=E_{i}*\Phi \left(E_{i}\right)$$

Φ(E_i_) being the differential photon fluence at energy E_i_ (photons cm^−2^ MeV^−1^) per initial source photon. A, *C* and *T* are conversion constants having numerical values, *B* = 1.602 × 10^−13^ J MeV^−1^, *C* = 10^−3^ kg g^−1^ and *T* = 3600 s h^−1^; *D* is the number of particles emitted per Bq and is equal to 2.364[14] for ^192^Ir and 0.851 for ^137^Cs.

For *F5 tallies, the relative errors R of the mean value are less than plus/minus 0.003% for 1 × 10^7^ particle histories considered in the BRIT LDR and vaginal cylinder simulations and are as shown Table in Tables 2 and 3 for other simulations.

**Table 2:** Dose rates at point – A, rectum and Bladder points computed for Fletcher Green LDR applicator

*Reference points*	*Dose Rate (cGy/h) (Sources only)*			*C_B_/C_M_
		
	*PENELOPE^(9)^*	*BrachyTPS(C_B_)*	*MCNP(C_M_)*	
Point - A	46.290	49.350	47.134 (± 0.0017%)	1.047
Rectum point	92.890	95.520	90.851 (± 0.0013%)	1.051
Bladder point	33.760	34.230	33.352 (± 0.0018%)	1.026

* C_B_/C_M_ = Ratio of BrachyTPS calculated value to MCNP calculated value

**Table 3:** Dose rates at point – A and point – B computed for FW HDR applicator

*Reference points*	*Dose Rate (Gy/h) (Sources only)*		*C_B_/C_M_
		
	*BrachyTPS (C_B_)*	*MCNP (C_M_)*	
Point - A	7.422	7.544 (± 0.0003%)	0.983
Point - B	2.509	2.633 (± 0.0002%)	0.953

* CB/CM = Ratio of BrachyTPS calculated value to MCNP calculated value

#### Reference Points for Dose Rate Computations

The two most commonly used systems for dose specifications in the treatment of cervical carcinoma are the Manchester system[1–3] and the ICRU[4] system. In the present study, the dose rate computations of intracavitary applicators are performed at points A and B of Manchester system and ICRU rectum and bladder reference points. Except for vaginal cylinder, in all other MCNP simulations, dose rates are computed only at the dose specification points. But in all BrachyTPS simulations, for each applicator, the dose distributions are computed in a 10 × 10 × 10 cm^3^ grid around the region of interest by taking into account each dwell position of the source.

## Results and Discussions

Discussions are aimed at comparison of BrachyTPS results with those of one of the most precise computational tools like MCNP code. The main cause of uncertainties involved in BrachyTPS calculations is extrapolation or interpolation of buildup factors used with the package and it leads to uncertainties up to five per cent. As this is expected in all BrachyTPS simulations they are not reported separately in each of the following discussions.

### Dosimetry of BRIT LDR Applicator

The dose rates at reference points are estimated for ideal positioning of applicators with the straight and tilted, long and medium IU tubes, using BrachyTPS and MCNP codes. The source assembly meant for long uterine tube has three source capsules (S_1_, S_2_ and S_3_) of nominal air kerma strengths 350, 230 and 230 *μ*Gy h^−1^ m^2^ (120, 80 and 80 mCi), from the fundus end and the medium uterine tube has two source capsules of nominal air kerma strengths 350 and 230 *μ*Gy h^−1^ m^2^ (120 and 80 mCi). The vaginal applicators have one source (S_4_ or S_5_) each of 230 *μ*Gy h^−1^ m^2^ (80 mCi). Sources are usually loaded in such a way that the fundus end of uterine tube has higher strength, CSA-1 type source. The closed end of the source (thickness one mm) faces the closed end of the applicator and the cap end of the source faces outward. The loading is similar for vaginal applicators with a single CSA-2 type source. The simulations have been performed for different cases with the separation between the vaginal ovoids varying from 2.5 cm to 4.0 cm and the distance of their centres below external OS level varying from 1.25cm to 2.0cm with the source loadings as specified in the previous studies.[15,16]

To study the applicator influence in the absorbed dose rate at the reference points, two different geometries are considered. One with the sources loaded in ovoids and tandem and the other with sources only, arranged in the same way as if they were inside the ovoids and tandem. The dose rates computed at point -A and point - B for long straight Intrauterine tube (IUT) and vaginal ovoids with and without applicator materials are given in Tables 4–5 details out point –A and point – B dose rates for various angulations of IUT and for various separations of vaginal ovoids of BRIT LDR applicator.

**Table 4:** Dose rates at point – A and point – B computed for BRIT LDR applicator

*Applicator*	*Source Position*	*Without Applicator*				*With Applicator*			
	
		*Dose Rate cGy/h at*				*Dose Rate cGy/h at*			
	
		*Point* – *A* (2.0, 2.0, 0.0)		*Point* – *B* (5.0, 2.0, 0.0)		*Point* – *A* (2.0, 2.0, 0.0)		*Point* – *B* (5.0, 2.0, 0.0)	
					
		*Brachy TPS*	*MCNP*	*Brachy TPS*	*MCNP*	*Brachy TPS*	*MCNP*	*Brachy TPS*	*MCNP*
	Fundus(S_1_)	28.0	28.982	9.74	10.213	27.6	28.574	9.59	10.149
Long Straight IUT	Middle (S_2_)	46.2	47.954	8.66	8.838	45.7	46.745	8.58	8.777
	Lower (S_3_)	50.9	50.857	9.16	8.920	49.1	50.510	8.96	8.907
	Total	125.1	127.794	27.56	27.970	122.4	125.829	27.13	27.833
	Right (S_4_)	18.9	18.544	9.51	9.306	18.6	18.295	9.32	9.253
Vaginal ovoids	Left (S_5_)	9.51	8.876	4.16	4.063	9.32	8.530	4.05	4.005
	Total	28.41	27.421	13.67	13.369	27.92	26.824	13.37	13.259
Long IUT & Vaginal Ovoids		153.51	155.214	41.23	41.339	150.32	152.654	40.50	41.091

**Table 5:** Dose rates (cGy/h) at point – A and point – B computed for various Intrauterine tube and Vaginal Ovoids of BRIT LDR applicator

*S. No*	*Applicator*	*IUT Angulation*	*Dose rates (cGy/h) at Point - A*		*C_B_/C_M_	*Dose rates (cGy/h) at Point - B*		*C_B_/C_M_
						
			*Brachy TPS(C_B_)*	*MCNP(C_M_)*		*Brachy TPS(C_B_)*	*MCNP(C_M_)*	
1	Long Intrauterine tube (IUT)	0°	122.4	125.829	0.973	27.13	27.833	0.975
		15°	122.4	125.875	0.972	27.13	27.865	0.974
		30°	122.4	125.956	0.972	27.14	27.937	0.971
		40°	122.4	125.961	0.972	27.14	27.888	0.973
2	Medium Intrauterine tube	0°	113.1	119.145	0.949	21.16	21.972	0.963
		15°	113.1	119.179	0.949	21.16	21.930	0.965
		30°	112.3	118.383	0.949	21.12	21.959	0.962
		40°	113.1	119.375	0.947	21.16	21.953	0.964
3	Vaginal Ovoids 1.5cm below OS level & 2.5cm separation	0°	28.2	27.5839	1.022	12.97	12.795	1.014
		15°	28.58	27.9167	1.024	13.08	12.909	1.013
		30°	29.82	29.4087	1.014	13.37	13.196	1.013
		40°	31.18	30.9272	1.008	13.67	13.536	1.010
4	Vaginal Ovoids 1.5cm below OS level & 3.0cm separation	0°	27.92	26.9028	1.038	13.37	13.166	1.015
		15°	28.34	27.5234	1.030	13.48	13.378	1.008
		30°	29.59	29.2956	1.010	13.8	13.640	1.012
		40°	30.95	30.8147	1.004	14.13	13.994	1.010
5	Vaginal Ovoids 1.5cm below OS level & 4.0cm separation	0°	27.1	25.7425	1.053	14.36	14.195	1.012
		15°	27.45	26.8307	1.023	14.5	14.341	1.011
		30°	28.67	28.5246	1.005	14.89	14.706	1.013
		40°	30.02	29.8995	1.004	15.3	15.141	1.011
6	Vaginal Ovoids 1.25cm below OS level & 3.0cm separation	0°	31.69	30.1953	1.050	14.23	14.076	1.011
		15°	32.07	31.1308	1.030	14.34	14.145	1.014
		30°	33.4	33.1026	1.009	14.64	14.484	1.011
		40°	34.86	34.703	1.005	14.95	14.831	1.008
7	Vaginal Ovoids 1.75cm below OS level & 3.0cm separation	0°	24.87	24.1571	1.030	12.56	12.367	1.016
		15°	25.22	24.5885	1.026	12.68	12.516	1.013
		30°	26.38	26.0781	1.012	13	12.827	1.014
		40°	27.64	27.4974	1.005	13.34	13.160	1.014
8	Vaginal Ovoids 2.0 cm below OS level & 3.0cm separation	0°	22.26	21.7827	1.022	11.8	11.698	1.009
		15°	22.59	22.1876	1.018	11.92	11.752	1.014
		30°	23.64	23.3185	1.014	12.24	12.081	1.013
		40°	24.8	24.7834	1.001	12.58	12.410	1.014

* C_B_/C_M_ = Ratio of BrachyTPS calculated value to MCNP calculated value

In MCNP simulations, the dose rate reductions due to applicator wall material are estimated to be 1.7% and 0.6% respectively at point-A and point-B whereas BrachyTPS computed reductions at these points are 2.1% and 1.8% respectively. The reason for this less significant effect in these simulations may be due to thin applicator wall material and ‘Z’ of the material of the applicator. However, it may be higher and significant if thick walled applicator made up of high ‘Z’ material is used. Probably this may be much more significant, while considering shielded ovoids. Rodriguez *et al* and de Almeida *et al*,[8,17] reported from their studies that the variation of dose due to the effect of the shielded applicator is 15.6% and 14.0% in the rectum and bladder respectively and 5.6% in the point of prescription ~point A.

**Figure 5:**
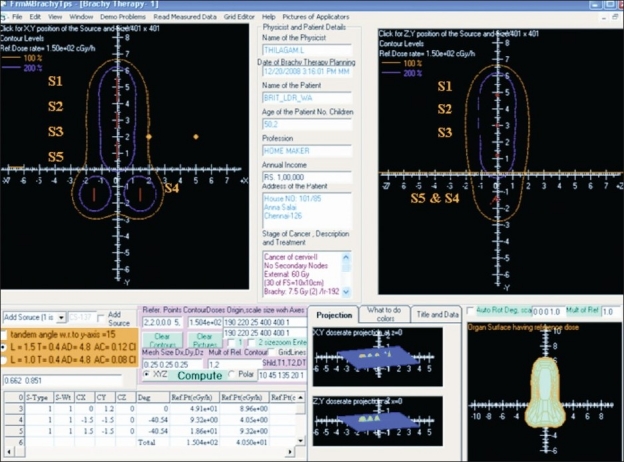
The 100 and 200% isodose distributions for BRIT applicator in the coronal and sagittal planes, normalized at point A and 3D isodose distributions

The absolute deviations of BrachyTPS computed dose values from MC results are observed to be within plus/minus 5.5% for BRIT LDR applicator. Dose distributions in the coronal and sagittal planes, normalized at point A, are obtained using BrachyTPS code for standard loading with long, straight intrauterine tube and vaginal ovoids (of angulation ~−40°) at three cm separation and is shown in Figure 5.

### Dosimetry of Fletcher Green Type LDR Applicator

The Fletcher Green type LDR applicator with Amersham made ^137^ Cs sources has been modeled as specified in the previous studies of Rodriguez *et al* and de Almeida *et al*.[8,17] In order to estimate the dose rate at the reference points, five CDC-J types, ^137^Cs (Amersham International) sources are considered. Two sources (S_4_ and S_5_) with the total linear reference air kerma rate of 72.3 *μ*Gy h^−1^ m^2^ cm^−1^ are modeled inside the colpostats of and three sources (S_1_, S_2_ and S_3_) with the total air kerma rate of 54.2, 36.2 and 36.2 *μ*Gy h^−1^ m^2^ cm^−1^ are modeled in the intrauterine tandem. In the present computations, only the sources have been modeled, arranged in the same way as if they were inside the applicator and tandem. The dose rates calculated by the BrachyTPS and MC codes are given in Table 2. The maximum deviations of BrachyTPS computed dose with the MC simulations for this applicator set is 5.1%.

**Figure 6:**
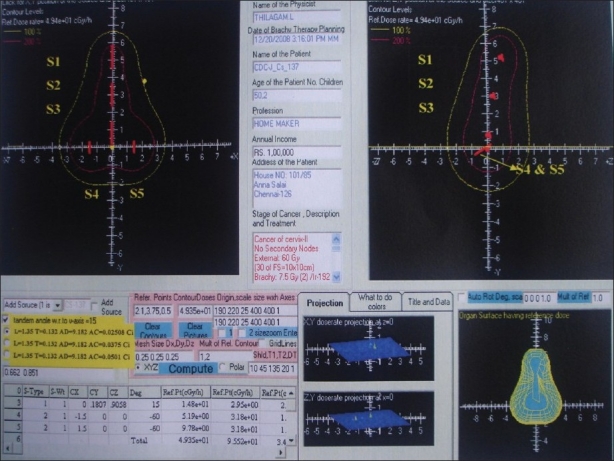
The 100 and 200% isodose distributions for Fletcher Green type LDR applicator in the coronal and sagittal planes, normalized at point A and 3D isodose distributions

Dose distributions in the coronal and sagittal planes, normalized at point A, are obtained using BrachyTPS code for standard loading with long, 15° tilted intrauterine tube and vaginal ovoids (of angulation 30° in relation to the handle) at three cm separation and is shown in Figure 6.

### Dosimetry of Fletcher-Williamson HDR Applicator

**Figure 7:**
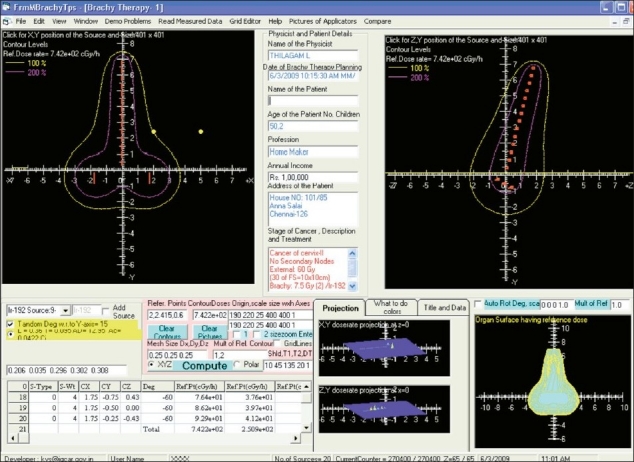
The 100 and 200% isodose distributions for Fletcher Williamsons HDR applicator in the coronal and sagittal planes, normalized at point A and 3D isodose distributions.

Computations have been performed for a conventional brachytherapy treatment plan for the HDR with the prescribed dose of 7.5 Gy at the average of left and right point-A. A total of 20 active dwell positions are used in the HDR plan computations, including 3 in each ovoid as in the available literature.[10] A 5-mm step size is used. The source specifications are used as reported in the previous study.[10] The dose rates calculated at the clinical reference points using the developed package and MC code are compared in Table 3. The maximum deviations of BrachyTPS computed dose with the MC simulations for this applicator set is −4.7%. Isodose distributions in the coronal and sagittal planes, normalized at point A, computed using BrachyTPS code is shown in Figure 7.

### Dose Distribution Around Shielded Vaginal Cylindrical Applicator Set

**Figure 8:**
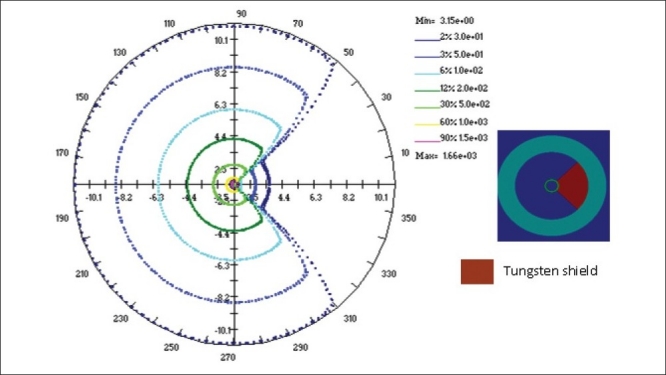
Dose rate distribution (cGy/hr) around a 3.0 cm diameter vaginal cylinder (with a 90° tungsten shielding) computed using MCNP4B and BrachyTPS codes

**Figure 9:**
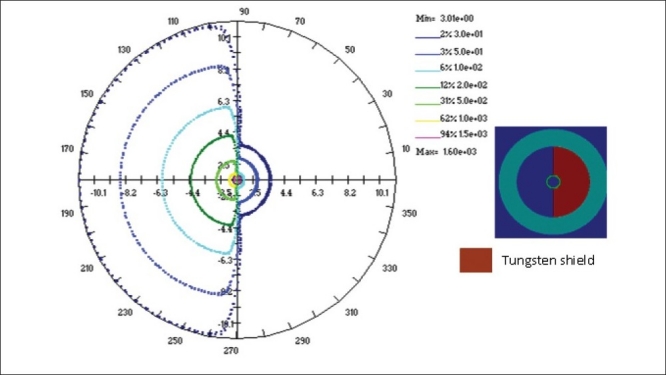
Dose rate distribution (cGy/hr) around a 3.0 cm diameter vaginal cylinder (with a 180° tungsten shielding) computed using MCNP4B and BrachyTPS codes.

**Figure 10:**
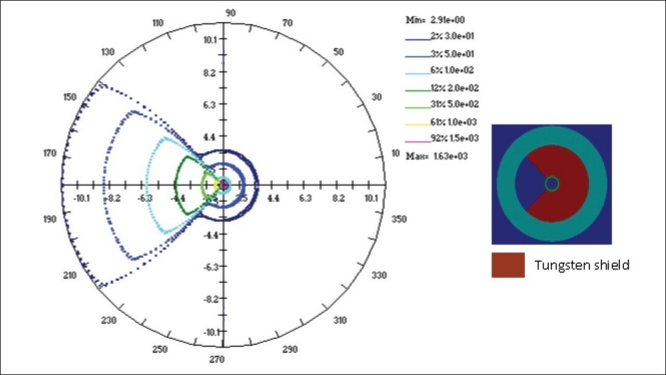
Dose rate distribution (cGy/hr) around a 3.0 cm diameter vaginal cylinder (with a 270° tungsten shielding) computed using MCNP4B and BrachyTPS codes.

The study has been carried out to obtain the dose rate distributions around the shielded vaginal applicator. The computations have been performed using BrachyTPS and MCNP codes under identical conditions for 0.8 cm thick 90°, 180° and 270° tungsten shielding. Dose rates are computed sequentially from 0° to 180° at every 5° interval for each radial distance starting from 1.5 cm, from the centre of the cylinder to 11.0 cm in steps of 0.5 cm. In MCNP simulations, the dose rates are computed only from 0° to 180° and then the dose distribution between 180° and 360° are duplicated from those of 0° to 180°. The 30, 50, 100, 200, 500, 1000 and 1500 cGy/hr isodose lines obtained from MCNP and BrachyTPS simulations are plotted for all the three cases using BrachyTPS software and are shown in Figures 8–10. The results computed using BrachyTPS code show good agreement (less than two per cent deviation) with the MCNP results in the unshielded region compared to the shielded region, where the deviations are observed to be up to five per cent. The deviations can be observed from the spread in the plots.

## Conclusions

Computational efficiency of the newly developed brachytherapy treatment planning package BrachyTPS with the dose calculation algorithm, accounting for heterogeneities exist in ICBT applicators is analyzed. Accuracy of the software is tested by performing dose rate computations for some ICBT applicators commonly used in the treatment of uterine cervical carcinoma and by comparing with estimates obtained from MC simulations and with the other available literatures. The following are the broad observations made:

- The developed point kernel code package BrachyTPS is capable of addressing the applicator shielding and its heterogeneities in dose calculations.
- The buildup factor taking care of scattering term hastens calculations using BrachyTPS whereas MC simulations are characterized by slow computational time. Therefore, the developed package may serve as a quick tool to compute and display dose distributions.
- In MCNP simulations of BRIT LDR applicator, the dose rate reductions due to applicator wall material are estimated to be 1.7% and 0.6% respectively at point-A and point-B whereas they are 2.1% and 1.8% respectively in BrachyTPS calculations. The reason for this less significant effect observed in these simulations may be due to thin applicator wall material and ‘Z’ of the material of the applicator. However, it may be higher and significant if thick walled applicator made up of high ‘Z’ material is used. Probably this may be much more significant, while considering shielded ovoids.
- The dose rates computed at clinical reference points of Manchester system and ICRU -38 by using BrachyTPS code, employing multiregional buildup factors proposed by Kalos[7] are found to be in good agreement with the MCNP results for all the cases studied and with the available literature[17] for Fletcher green type LDR applicator.
- The percentage deviations of BrachyTPS computed dose rate values from the MC results are observed to be within plus/minus 5.5% for BRIT LDR applicator, found to vary from 2.6% to 5.1% for Fletcher green type LDR applicator and are up to minus 4.7% for Fletcher-Williamson HDR applicator.
- The dose rate distributions computed around the shielded vaginal applicator using BrachyTPS code agree well (less than two per cent deviation) with the MCNP results in the unshielded region compared to the shielded region, where deviations are up to five per cent.
- The 2D isodose distributions, computed using BrachyTPS code in coronal and sagittal planes, normalized at point A also agree well with the available literatures.[10,15,16]

The present study throws light on suitability of point kernel dose calculation techniques for brachytherapy treatment planning and the developed package is observed to meet the clinical requirements for precision and time.
